# Supplementary material for: Sustainable care: How CSR shapes wellbeing in healthcare organizations in Beijing, Shanghai, and Guangzhou
Source: PLoS One. 2025 Feb 25;20(2):e0316601. doi: 10.1371/journal.pone.0316601 (PMC11856263; doi:10.1371/journal.pone.0316601)
Supplement: Attachment — Submitted filename: Reviewers comments and author responses 1.docx [file pone.0316601.s004.docx]

1. Please ensure that your manuscript meets PLOS ONE's style requirements, including those for file naming. The PLOS ONE style templates can be found at
   <https://journals.plos.org/plosone/s/file?id=wjVg/PLOSOne_formatting_sample_main_body.pdf> and 
   <https://journals.plos.org/plosone/s/file?id=ba62/PLOSOne_formatting_sample_title_authors_affiliations.pdf>

**Response:** Thank you for the guidance, we have followed th above guidelines during the revision stage of our manuscript.

2. Thank you for submitting the above manuscript to PLOS ONE. During our internal evaluation of the manuscript, we found significant text overlap between your submission and previous work in the [introduction, conclusion, etc.].
    
   We would like to make you aware that copying extracts from previous publications, especially outside the methods section, word-for-word is unacceptable. In addition, the reproduction of text from published reports has implications for the copyright that may apply to the publications.
    
   Please revise the manuscript to rephrase the duplicated text, cite your sources, and provide details as to how the current manuscript advances on previous work. Please note that further consideration is dependent on the submission of a manuscript that addresses these concerns about the overlap in text with published work.
    
   [If the overlap is with the authors’ own works: Moreover, upon submission, authors must confirm that the manuscript, or any related manuscript, is not currently under consideration or accepted elsewhere. If related work has been submitted to PLOS ONE or elsewhere, authors must include a copy with the submitted article. Reviewers will be asked to comment on the overlap between related submissions (<http://journals.plos.org/plosone/s/submission-guidelines#loc-related-manuscripts)>.]
    
   We will carefully review your manuscript upon resubmission and further consideration of the manuscript is dependent on the text overlap being addressed in full. Please ensure that your revision is thorough as failure to address the concerns to our satisfaction may result in your submission not being considered further.

**Response:** Thank you again for the detailed feedback on the above matter. Please be informed that while revising our manuscript, we have significantly considered the above issue. We ensure that no part of the revised manuscript includes the text extracted and reported from the previous research without any modifications. To further avoid any sort of ambiguity, we have also uploaded a similiarity report generated by Turnitin Software. As you can see the attached document (supplementary file) does not report any of such issues in our revised manuscript. However, if this journal is using another software for detecting similarity issue (which is not in our notice), and if our work is still flagged with plagiarism issue, we request you to kindly share this report with us, so that we can further address this issue. Hopefully, you will realize our concern and stance on this matter. Best Regards

3. We note that your Data Availability Statement is currently as follows: [All relevant data are within the manuscript and its Supporting Information files.]

Please confirm at this time whether or not your submission contains all raw data required to replicate the results of your study. Authors must share the “minimal data set” for their submission. PLOS defines the minimal data set to consist of the data required to replicate all study findings reported in the article, as well as related metadata and methods (<https://journals.plos.org/plosone/s/data-availability#loc-minimal-data-set-definition>).

For example, authors should submit the following data:

- The values behind the means, standard deviations and other measures reported;
- The values used to build graphs;
- The points extracted from images for analysis.

Authors do not need to submit their entire data set if only a portion of the data was used in the reported study.

If your submission does not contain these data, please either upload them as Supporting Information files or deposit them to a stable, public repository and provide us with the relevant URLs, DOIs, or accession numbers. For a list of recommended repositories, please see <https://journals.plos.org/plosone/s/recommended-repositories>.

If there are ethical or legal restrictions on sharing a de-identified data set, please explain them in detail (e.g., data contain potentially sensitive information, data are owned by a third-party organization, etc.) and who has imposed them (e.g., an ethics committee). Please also provide contact information for a data access committee, ethics committee, or other institutional body to which data requests may be sent. If data are owned by a third party, please indicate how others may request data access.

**Response:** We have uploaded the raw data file as supplementary file during the reviosn submission.

4. PLOS requires an ORCID iD for the corresponding author in Editorial Manager on papers submitted after December 6th, 2016. Please ensure that you have an ORCID iD and that it is validated in Editorial Manager. To do this, go to ‘Update my Information’ (in the upper left-hand corner of the main menu), and click on the Fetch/Validate link next to the ORCID field. This will take you to the ORCID site and allow you to create a new iD or authenticate a pre-existing iD in Editorial Manager. Please see the following video for instructions on linking an ORCID iD to your Editorial Manager account: <https://www.youtube.com/watch?v=_xcclfuvtxQ>

**Reviewers' comments and Authors Responses**

**Reviewer #1: Introduction**

This introduction covers the fundamental aspect of mental health of employees in general, and the specific angle of burnout, and their effect on the overall success of the organization, particularly in the healthcare sector. The author has clearly delineated how the relationship between employee well-being and organizational performance is paramount in healthcare organizations and that addressing burnout is a crucial consideration. The idea that CSR initiatives and their potential effects on curbing employee burnout while as well as in line with SDGs is a novel and timely phenomenon. This approach therefore not only makes CSR more holistic but also strategically positions it to complement the global sustainable development agenda.
Yet, I suggest that the first part should be more precise in defining key notions such as CSR and burnout so that the readers with different backgrounds can grasp the same meaning. Furthermore, although the paper does well to set the research agenda and its applicability to healthcare in China, it would be more useful if it had a section that briefly discusses the research methodology or the theoretical framework to be employed in the study. Such arrangement will help readers to understand the mechanisms through which the research objectives will be attained and how the proposed moderators and mediators) will be investigated in the context of CSR-employee burnout. Moreover, due to the fact that the effect of CSR on employee outcomes is multi-faceted, illustrating case studies of CSR initiatives that have succeeded in reducing burnout would make the argument more convincing. To end the paper, there should be a discussion about the limitations of literature currently existing and how this study aims at addressing these gaps, in order to provide a clearer framework of how this study has made a contribution to the field.

**Response:** Your valuable advice is much appreciated. Thus, we have introduced several changes concerning the introduction section as per your suggestions. To begin with, some changes have been made to the introduction to clarify the meaning of the key concepts, for example, Corporate Social Responsibility (CSR) and burnout. This addition can be considered as an attempt to guarantee that the given text will be comprehensible to the audience with various levels of knowledge about the principles of our research. The definitions are put immediately after the first paragraph of the introduction section. Secondly, in order to maintain the structure of the paper that can be easily followed, we provide short information on the research methodology and the theoretical framework used in this study. This addition is inserted before the final paragraph of the introduction section and it outlines the Positive Psychology Theory (PPT) that informs the research as well as the Structural Equation Modeling (SEM) employed in the analysis of data. This part is intended to provide the readers with a clear vision of how the research objectives will be accomplished. Finally, we have broadened the discussion by elaborating on the various ways through which CSR influences employee outcomes, as well as stressing on the significance and usefulness of CSR in the sphere of healthcare. Also, we have integrated a section outlining the limitations of prior studies and how our research fills those research gaps. This section is located before the paragraph that describes the organization of the paper and is intended to offer a clearer framework for our study’s potential contribution to the literature. It is with these changes in mind that it is hoped the introduction will be more concise and include all the necessary information that will be required in the subsequent sections of the paper.
Literature

The hypotheses section is presented in a flawless manner and it suggests that CSR can directly contribute to employees' well-being, such as lowering burnout, increasing happiness and promoting psychological safety. The positive organizational outcomes of the integration of CSR is well-studied, with a detailed examination of how CSR initiatives can create a more innovative and less-pressure work environment. The theory of positive psychology (PPT) and the PERMA model have been integrated into the discussion, which gives an outline to the hypotheses with a theoretical aspect.
Nevertheless, the hypotheses are well-built but there needs to be more focus on the empirical evidence substantiating the relationships. Similarly, cited studies that have looked at the direct CSR effects on burnout, happiness, and psychological safety would add more value to the arguments. On top of that, though altruism is considered as a moderating factor, the description of how and why altruism affects the connection between CSR and employee outcomes should have been further clarified. For this, it would be good to include more illustrative examples or case studies that demonstrate how altruism in the workplace multiplies the positive outcomes of CSR.
**Response:** Thank you for your valued suggestions. To support the relationships between CSR and the dependent variables, we have provided more research findings. For instance, research indicates that CSR practice reduces a high level of burnout due to supportive workplace practices. CSR activities improve the level of happiness of the employees due to the fact that the organizational and personal values are more congruent, thus providing meaning and purpose. CSR practices also positively impact employees’ psychological safety since it create ethical work environments that are also diverse. We provided further explanations and examples regarding the use of altruism as a moderating factor with the help of several case studies. Altruistic employees gain more benefits from CSR activities because such activities match their desire to be of help to others and make them feel psychologically safe. For example, a research proved that in organizations where the level of altruism is high, CSR activities positively affect the level of happiness and decrease burnout of the workers. These additions enrich the arguments and give a clear and complementary picture of the relationships explored. Hopefully, you will like our revision efforts. Thanks again.

Methods

The Ethical and detailed Methods section has a well-grounded selection of Beijing, Shanghai and Guangzhou as the representatives of health care system of China. The prospective design, which is multifaceted and incorporates data from three waves, enriches the opportunity of observing the changes, and, therefore, adds validity to the findings. The application of existing scales provides an indication of the credibility of the data collection process.
Nevertheless, the methodology of the study should be enriched with the explanation of the reasons for the two week gaps between the survey waves and a thorough discussion on the possible sources of non-response bias. Although SEM model is mentioned, it would be better to detail the SEM model specifics for better understanding.

**Response:** I appreciate your input in this research project. We have enhanced the methodology section by providing justification for the two week interval between the survey waves; the purpose was to reduce the likelihood of recall bias and to capture any temporal shifts in attitude and perception. This design let us to investigate dynamic process of employees’ interaction with CSR initiatives and their outcomes. We have also had a detailed discourse on sources of non-response bias and ways of dealing with it, including follow up reminders, and anonymity of the respondent. Additional information regarding the SEM specifics has been provided to increase comprehensibility. To ensure the validity of the constructs and the hypothesized relationship, we used a rigorous SEM model, which includes measurement model and structural model.

Discussion


The dialogue strongly links CSR with the elimination of employee burnout, happiness, and psychological safety in the Chinese healthcare area, which is in line with the positive psychology theory. The study focuses on the two mediators – happiness and psychological safety – and the moderator altruism which makes the CSR-burnout relationship more understandable. The insights gained can be used to improve individual and organizational dynamics affecting employee wellbeing.
While the conceptual and practical issues are well explained, a detailed analysis of the challenges of CSR implementation would give more depth to the topic being discussed. The limitation of the study such as the focus on urban areas and the cross-sectional design should be noted. Future research can fill the gaps by exploring the effects of CSR in rural areas, using longitudinal designs, and taking into account cultural factors in order to broaden the understanding.

**Response:** Thank you for your detailed feedback. We have added a detailed analysis of the challenges of CSR implementation and noted the limitations of our study, including the focus on urban areas and the cross-sectional design. We are positive that you will like our revised efforts. Best Regards

**Reviewer #2:**

This manuscript investigates the interconnection between corporate social responsibility (CSR) perceptions and employee burnout using data related to the healthcare system of China. This research explores the linkages between CSR initiatives, employee burnout, happiness, psychological safety, and altruistic behavior. Data on CSR perceptions, employee burnout levels, happiness, psychological safety perceptions, and altruistic motivations were collected through a thrice-administered questionnaire with a sample size of 392 participants from three cities. The author concluded that CSR practices cause burnout reduction, while happiness and psychological safety of workers are mediators of this interconnection. This original research highlights the impact of CSR perceptions on employee well-being and sustainable human capital development.

With great respect to the purpose of the job and agreement with hypothesis tested in this research, there are several methodological and theoretical concerns that can be considered for further improving the quality of this research.

1. Data collection and sample design.
1.1. CSR measurement scale.
According to the article, “the survey contained Turker’s [34] six items CSR scale, with items such as “my hospital aims at protecting and enhancing environment” (p. 8, para 1). However, according to Turker (2009) there are 4 factors measured through 18 questions. More information is required about shortened list of questions used for CSR measurements. Specifically, it is important which of the four factors identified by Turker (2009) were measured (CSR to social and nonsocial stakeholders (1), employees (2), customers (3), government (4)).

**Response:** Thank you again for the valued input. The scale developed by Turker (2009) comprises four dimensions of CSR, which is served by 18 questions, for social and nonsocial stakeholders, employees, customers, and government. For this study, we employed a shortened version of the scale composed of six questions that focused on the overall CSR practice of an organization. This decision was made to reduce the length of the survey and to focus more on the general view of CSR instead of it’s many facets. Prior research has effectively employ such a strategy to scale down the number of items used in evaluating CSR commitment (for more detail, kindly see the revised text of our manuscript). This method has proven to be efficient in identifying the core concept of CSR without compromising on the efficiency of the survey and the attentiveness of the respondents. By looking at overall CSR participation, our intention was to capture the broad effect of CSR on the employees as it was in line with our research goals.
1.2. CSR related terminology.
According to Turker (2009) their scale reflects CSR perceptions of employees as internal stakeholders of a business. This does not contradict the purpose of current research but requires using corresponding wording in the text. E.g., the first hypothesis tested is formulated as follows:
H1: The extent of an organization’s CSR engagement is directly associated with low levels of employee burnout. (p.5, para 2). While in fact, this should be about the level of employee awareness about the organization’s CSR engagement.

**Response:** Thank you for your feedback. We have revised the wording of our first hypothesis to accurately reflect your above concern. Hopefully, you will appreciate this revision.

1.3. Sample design.
“The study focused on three key cities such as Beijing, Shanghai and Guangzhou whose selection was based on their strategic positioning in China’s healthcare landscape as political economic, and health innovation hubs. In terms of medical policy formation and reform, Beijing is among the leading cities. Shanghai exhibits the most advanced medical technologies through its role as a financial center while at the same time experiencing significant growth of private hospital industry. For a long time Guangzhou has been famous for medical researches and educational institutions promoting development of medical standards and practices. This selection of cities offers a holistic picture of the CSR practices in various healthcare contexts in China, improving the study's validity and generalizability. Hospitals in these listed cities which had visible CSR plans were the target for data collection in order to establish the link between CSR initiatives and employee outcomes.” (p.7, para 3)
According to the author, the selection or participant organizations was made based on the major criteria of CSR programs to be well-developed, advanced, and recognized. However, to expand conclusions for the whole healthcare industry as stated in the title of the article, the sample should include organizations with different levels of CSR engagement.
It is worth changing the title to avoid unreasonable level of generalization.

**Response:** Three cities namely Beijing, Shanghai and Guangzhou were chosen for this study because of their political economic and health innovation importance in China. In regards to the formation and development of medical policies and reforms, Beijing can be considered as one of the most advanced. Shanghai demonstrates the most cutting-edge technologies in the medical field by virtue of being the financial hub and at the same time, witnesses the booming of the private hospitals. Ever since the ancient times Guangzhou was known for medical researches and educational institutions encouraging the growth of medical facilities and prognosis. The selection of these cities provides an overall view of the CSR activities in different contexts of the healthcare sector in China and thus increases the validity of the study and the generalizability of the findings. Since the aim was to have a comprehensive analysis of CSR, the participant organizations were selected based on the existence of organized, well-developed, advanced, and recognized CSR programs. However, we admit that this criterion might limit the generalization of the findings to the whole healthcare industry. In light of this, it is recommended that future studies incorporate organizations that have different degrees of CSR involvement to get improved insights. Moreover, we have also revised the title of our study. Hopefully, you will like our revision efforts. Regards

2. Theoretical approach
While investigating possible psychological factors mediating and moderating the interconnection between CSR perceptions and burnout, the author suggests happiness and psychological safety to be independent mediators based on the literature review. However, there is plenty of information about high level of correlation and even causality between these two factors (e.g., Plester & Lloyd, (2023); Wang, Kang & Bong (2022)).
This requires some modifications of measurement model including connections between mediating factors or even sequential mediation as proposed in Wang, Kang & Bong (2022).
This is also visible through current research results (see Table 4: Discriminant validity, p.12). According to the table, the level of correlation between happiness and psychological safety is 0.527.

**Response:** We would like to thank you for your valued and detailed feedback concerning the possible interaction between happiness and psychological safety as the mediators of the connection between CSR and burnout in our study. Although, we understand that there exists a strong empirical literature suggesting the association of these factors and even a causality as evidenced by Plester & Lloyd (2023) and Wang, Kang & Bong (2022) respectively, our decision to treat happiness and psychological safety as mediating variables is deliberate and methodologically sound. Specifically, our study utilizes partial least squares structural equation modeling (PLS-SEM) through the software SMART-PLS, and it is based on a different research logic from the covariance-based structural equation modeling (CB-SEM) approach such as AMOS. PLS-SEM focuses on the highest proportion of variance in the dependent variables explained by the model’s independent variables through its R² values rather than model fit indices. This approach is most beneficial in cases where the major goal of the research is to provide an empirical analysis of the constructs and their relationships, as opposed to testing a specific theoretical model.

In PLS-SEM, estimation of path coefficients, weights as well as the loadings is done through an iterative algorithm, which gives the best estimate of the variance in the data. This method enables one to establish the fitness of the models with several indicators and constructs even if the sample is small or the data is not normally distributed. Also, PLS-SEM provides estimation of measurement model between constructs and indicators and the structural model between constructs at the same time, thus, the results of PLS-SEM are much more accurate than those of EFA and CFA since it controls the measurement errors and gives a correct representation of the constructs. SMART-PLS is also quite capable to handling multicollinearity among the predictor constructs, which means that Highly correlated constructs can be included in the model without affecting its validity. This capability is very useful for our study since in this case happiness and psychological safety are two different but conceptually linked constructs. Thus, by perceiving these variables as distinct constructs, it becomes possible to determine the extent to which they are independent predictors of burnout while adhering to the chosen theoretical framework and maintaining measurement clarity.

The correlation of 0.527, which is the correlation found in the results between the level of happiness and psychological safety, can be considered as moderate. However, this does not mean that there is a causal relationship between them or that there is a need for a sequential mediation model. Rather, it only emphasizes how these constructs are different but are not mutually exclusive. Thus, we retain their theoretical distinctiveness and, therefore, methodological purity as separate variables facilitating the analysis of the effect of each factor on employee burnout. We hope you understand our stance on this particular matter. Thanks again for a kind and important feedback on our work. Best Regards

3. Statistical analysis.
The structural equation modeling (SEM) used for confirming the measurement model and research hypothesis assumes testing the extent to which a hypothesized model provides an appropriate characterization of the collective relationships among research variables. For this purpose, researchers must assess the “fit” between the model and the sample’s data.
There is no data on model fit in the results section of the article. CFI, TLI, GFI, or RMSEA indices are recommended for model equivalence-testing to test and compare goodness of fit for structural equation models (Peugh & Feldon, 2020). Further analysis of variable interconnections in Table 5: Hypothesis results (p.13) is not informative without analysing model fit.
**Response:** For this point, we humbly refer to our previous point as SMART-PLS does not produce indices like CFI, TLI, GFI, or RMSEA and uses composite reliability, average variance extracted (AVE), and path coefficients to assess model quality, ensuring robust and predictive results aligned with our research objectives. Thanks again for the feedback.

4. Proofreading.
Some additional proofreading can be recommended. E.g., “In addition to corroborating the empirical evidence of the critical roles of happiness and psychological safety in improving workplace wellbeing, these findings also suggest that organizations should aim to create settings that facilitate such positive psychological states throughout the fight against burnout” (p.15, para 1).
**Response:** Thank you for the keen observation. We ensure, that in the revised manuscript, you will not find any of such inaccuracies.

References
Peugh J, Feldon DF. (2020). How Well Does Your Structural Equation Model Fit Your Data? Is Marcoulides and Yuan's Equivalence Test the Answer? CBE Life Sci Educ. Sep;19(3):es5. doi: 10.1187/cbe.20-01-0016. PMID: 32510273; PMCID: PMC8711809
Plester, B. A., & Lloyd, R. (2023). Happiness Is ‘Being Yourself’: Psychological Safety and Fun in Hybrid Work. Administrative Sciences, 13(10), 218. <https://doi.org/10.3390/admsci13100218>
Wang,W., Kang, S., Bong, S. (2022). Servant Leadership and Creativity: A Study of the Sequential Mediating Roles of Psychological Safety and Employee Well-Being. Front. Psychol., Sec. Organizational Psychology, Volume 12, <https://doi.org/10.3389/fpsyg.2021.807070>

**Reviewer #3:**

Here are some major comments to enhance the clarity, depth, and impact of your article:
1. The introduction section need again major revisions for example break introduction in sub-sections if possible.

**Response:** Thanks for the valued input. We have revised the introduction part significantly. Moreover, we have also divided it into different sub-sections.
2. Please revise the novelty of this work in the introduction section and if possible please extend the introduction section up two or three paragraph.

**Response:** We appreciate your input and feedback on our manuscript. We have worked to improve the framework of the introduction section where we state the purpose of this research and explain how the study is unique. Particularly, we have enriched the introduction section by adding more text and paragraphs to give more background information for the connection between CSR and employee wellbeing, with the focus on the healthcare industry. To the present study, we have added recent investigations as well as deepened on the sophisticated relation between CSR actions and the prevention of burnout, underlining the connection with SDGs. By making these changes, we seek to emphasize the novelty of our approach and the findings of our research in relation to prior publications. Hopefully, you will like our revised efforts.
3. Furthermore, if possible at the end of introduction section please add a line about the structure of the paper or rest of the paper section or style.

**Response:** Following your valued suggestion, we have added a concluding paragraph to the introduction section that outlines the structure of the paper
4. Please add the Literature review section separately from Introduction, I am suggesting to start the discussion with based on the theory. Which theory of behavior have you applied in this research such as; RBV is enough to cover your all details of variable and does it cover all aspect of variable? If yes please response with answer and if no please use the relevant theory in the update file.

Response: In order to make the manuscript more sequentially coherent, we have included a separate Literature Review section to give the reader a clear concept on the theoretical framework and prior research studies that have informed our work. The Positive Psychology Theory (PPT) has been retained as the underpinning theory since it is comprehensive in addressing all aspects of the variables. PPT encompasses only the pleasant aspects of human life and their well-being, which are positive emotions, engagement, relationships, meaning, and achievements (PERMA) to foster well-being. This theory has a lot of relevance to CSR objectives and is especially suitable for stressful sectors such as health. Thus, combining PPT with CSR studies allows getting a broader understanding of how CSR can help reduce burnout and improve employees’ quality of life.
5. Regarding literature review my suggestion is support your analytical writing with update references.

**Response:** Thanks again for the kind guidance, we have updated our Literature review section by including more up-to-date references. Thanks

6. If possible please add Conceptual Research Framework. Clearly mention the relation among all the variable. Please also mention IV, DV, mediation and moderator variable.

**Response:** Following your valued suggestion, we have now included the conceptual model (Revised Figure 1), in the revised version of our manuscript.
7. Could you please clearly write the methodology section again and why you have only selected these three cities such as; Beijing, Shanghai and Guangzhou Page number 7?

**Response:** Your valued suggestion is much appreciated. In the revision, we have enhanced the section on the methodology through providing more details on why Beijing, Shanghai, and Guangzhou have been chosen. These cities were selected based on their roles in China’s health system as political, economic, and health technology centers. Beijing is the capital leading in the making and reform of medical policies, Shanghai is famous for medical advanced technology and the rapid development of the private hospitals, Guangzhou has a long history in medical research and educational center. This selection gives a comprehensive view of CSR practices in various heath care settings, thus increasing the generalizability of the study. Moreover, we have divided the methods section in different sub-division for a better and smooth readibilty. Last, but not the least, the methods section has also been revised in different part. Hopefully, the revised Methods section will satisfy you.

8. Please also add a table about the variable and its operational definitions, and its source from where it is adopted. Please clarify the research design, data collection methods, and analysis procedures. Ensure that your methodology is robust and suitable for addressing the research question.

**Response**: Thank you for the valued input. We have added a table summarizing the variables, their operational definitions, and sources to clarify the research design, data collection methods, and analysis procedures.
9. How authors have measure the possibility of multicollinearity among latent variables.

Response: Thank you for your comment. To assess multicollinearity among latent variables, we calculated the Variance Inflation Factor (VIF) for each construct. All VIF values were below the threshold of 5, indicating that multicollinearity is not a concern in our model. We have now included these values in our revised Table 4 (Kindly see the last column). Regards

10. Please clearly update discussion and conclusion sections.

**Response:** Thank you again for sharing your valued thoughts with us. We have expanded the discussion and conclusion to reflect on its significance to the findings, connect them with prior research, and consider the general implications.
11. Expand the discussion section to thoroughly interpret and explain the results. Relate your findings to existing literature and discuss their broader implications and focus on page number 9.

Response: We again appreciate your suggestion. The section on Discussion has been extended to provide adequate analysis and interpretation of results, integration with the prior research, and implications of the study. This involves incorporating literature reviews and studies together with an extensive assessment of the results.

12. Revise the conclusion to summarize key findings and highlight their significance.

**Response:** Following your recommendations, we have revised the conclusion to summarize key findings and highlight their significance.
