# Supplementary material for: Sustainable care: How CSR shapes wellbeing in healthcare organizations in Beijing, Shanghai, and Guangzhou
Source: PLoS One. 2025 Feb 25;20(2):e0316601. doi: 10.1371/journal.pone.0316601 (PMC11856263; doi:10.1371/journal.pone.0316601)
Supplement: Attachment — Submitted filename: pone.0316601.docx [file pone.0316601.s006.docx]

**Reviewer #1:**

The Authors have done a decent job in revising their manuscript and in addressing my concerns. Congratulatoins.
I have no further comments, and hence, recommend the publication of this work in its current form.

Best of Luck to the authors

**Response:**

Dear Reviewer, Thank you for the kind evaluation and for appreciating our revised efforts. Best Wishes.

**Reviewer #2:**

I appreciate the job done to address methodological and theoretical concerns expressed in my primary review. Though some of the recommendations were considered, not all the necessary corrections were made.

1.Data collection and sample design.
1.1. CSR measurement scale.
The revisions were made to explain the shortened scale for measuring CSR perceptions.

**Response:** Thanks for the time and efforts again to evaluate our revised efforts. Thanks again for agreeing with our selection of short scale. Best Regards

1.2. CSR related terminology.
'According to Turker (2009) their scale reflects CSR perceptions of employees as internal stakeholders of a business. This does not contradict the purpose of current research but requires using corresponding wording in the text. E.g., the first hypothesis tested is formulated as follows:
H1: The extent of an organization’s CSR engagement is directly associated with low levels of employee burnout. - p.5, para 2
While in fact, this should be about the level of employee awareness about the organization’s CSR engagement.'

Some Hypotheses were revised, however, there are still many cases of using ‘CSR’ instead of ‘CSR perceptions’ in all the parts of the article text, as well as models, tables, etc.

E.g.,
H5: There exists a mediating mechanism of psychological safety in the CSR-burnout relationship – p.7 para 3
Psychological safety perceptions from the employees can act as a mediator in the relationship between CSR and burnout – p.7 para 3
H6: Altruism moderates the relationship between CSR and employee happiness, such that the positive effect of CSR on employee happiness is stronger among employees with higher levels of altruism - p.9 para 1
H7: Altruism moderates the relationship between CSR and psychological safety, such that the positive effect of CSR on psychological safety is stronger among employees with higher levels of altruism - p.9 para 1
The Conceptual model of this study is shown in below Figure 1 – p.9 para 1
Accordingly, the last wave entailed the measurement of the employee’s burnout (EB) so that the 11 relationships between CSR, altruism, happiness, psychological safety, and burnout could be examined over time. – p.11para 1
Table 6 presents a structural analysis of our proposed relationships as hypothesized in the
conceptual model of our research. CSR affects EB directly (-0.098) at p=0.008 level; CSR also
influences HP (0.158) and PS (0.232) significantly both having a p-value =0.000, indicating its
positive effect on these variables. Similarly, HP and PS also significantly predicted EB (-0.163
and -0.178 with p values <0.05). The CSR -> HP -> EB pathway reveals a mediation effect (-
0.026) by positioning HP as a mediator. PS's relationship with EB is also significant (-0.178, pvalue: 0.022), and the path CSR -> PS -> EB confirms PS as another mediator with a coefficient
of 0.041. The path CSR*AL1 -> HP -> EB is also significant (0.037, p-value: 0.006), showing
that AL alters the connection linking CSR and EB via HP. As well as, the relationship between
CSR*AL2 and PS is also significant (0.055, p-value: 0.002); it means that AL moderates through
PS. Figure 3 below represents our structural model showing various relationships – p.17 para 3
In our present study, we provide empirical evidence about CSR, employee burnout, happiness, psychological safety, and altruism dynamics within the healthcare sector in China – p.18 para1
We examine the 22 direct association of CSR with employee burnout, and how happiness and psychological safety mediate this relationship, as well as altruism moderating it – p.21 para1
Secondly, we establish that happiness and psychological safety could serve as mediators between CSR and burnout - – p.21 para1
Our research tried to bridge the gap between theoretical concepts and practical implications within the context of healthcare through an application of CSR principles within a global SDG framework as far as China is concerned. The present study has examined how certain concepts such as altruism, happiness, job burnout, and psychological safety are related to CSR – p.23 para 3
Consequently, the present research establishes an overall understanding of the significant relationship between CSR and employee health in the Chinese healthcare industry. – p.24 para 1

**Response:** Really thanks for highlighting the importance of using the proper wording in academic writing. We again acknowledge your efforts in evaluating our work. We also realize that you have spent significant amount of time in reading each line of our work. This is something that we truly appreciate and acknowledge.

We have revised our entire manuscript to carefully check where should we use CSR perceptions or CSR. Hopefully, in our latest revised work, you will not detect any of above inconsistency. Best Regards

1.3. Sample design.
Addressed through changing the title.

**Response:** Thanks again for the positive feedback.

2. Theoretical approach

'While investigating possible psychological factors mediating and moderating the interconnection between CSR perceptions and burnout, the author suggests happiness and psychological safety to be independent mediators based on the literature review. However, there is plenty of information about high level of correlation and even causality between these two factors (e.g., Plester & Lloyd, (2023); Wang, Kang & Bong (2022)).
This requires some modifications of measurement model including connections between mediating factors or even sequential mediation as proposed in Wang, Kang & Bong (2022).'
Reviewer comment: Partially addressed through explaining the purpose of investigating independent impact of happiness and psychological safety.
However, in my opinion, this can significantly reduce the quality of the model, which the author cannot evaluate due to limitations of the software used. It is strongly recommended to discuss possible modifications of the exploratory model after collecting more data and increasing the sample size.

**Response:** Thank you very much for your very meaningful comment on the very high correlation and potential causal connection between happiness and psychological safety and for the recommendation to continue investigating these relationships using sequential mediation or other advanced models. The Limitations and Future Research Directions section in our revised manuscript reflects this point carefully. In the revised manuscript, we have made explicit that this decision to model happiness and psychological safety as independent mediators was based on both theoretical and methodological grounds. Happiness is about emotional well-being and psychological safety is about interpersonal and environmental dynamic, which we acknowledge through the interconnection of these constructs as found in previous studies (Plester & Lloyd, 2023; Wang, Kang, & Bong, 2022).

Further, we recognize the utility of investigating sequential mediation or other complicated relationships to represent the intricate interplay among such mediators. Yet at this point, the data is already collected and analyzed, therefore extending the dataset is not possible. Considering that the current sample size and the use of PLS-SEM (a technique tailored for variance-based modeling) are involved, we aimed at providing robust and interpretable results within the current scope.

We have now pointed out this issue in the Limitations section, and suggested possible future research directions to deal with this. Specifically, we recommend future studies to:

a) To evaluate more complex models, we will need to collect datasets that are larger than what we have to date.
b) Using more advanced structural equation modeling techniques like covariance based SEM or Bayesian SEM test sequential mediation frameworks and bidirectional effects.
c) Explore further the causal pathways between happiness and psychological safety in different organizational contexts.

We think the additions we have made to the manuscript address both these concerns, and more generally, improve the rigor and transparency of our study. Although we have already given valuable insights based on the given dataset, we acknowledge the need for iterative research and continue to commit to further developing this foundation in future work.

Once again, we want to thank you for your valuable suggestions which contributed so much to the quality, if not also the direction, of our research.

Also, the ambiguous statements about Happiness and Psychological safety being independent factors, should be excluded from the article. These are just assumptions and limitations of tested exploratory model, rather than measurement results. Thus, they cannot be discussed as scientifically proven findings of this research. So, I suggest excluding the following:
Happiness and psychological safety are two separate variables that when added together can work independently to act as moderators of burnout – p.24 para 2

**Response:** Following your valued suggestion, we have removed the above lines from our revised manuscript. Thanks again for the feedback.

3. Statistical analysis.
The structural equation modeling (SEM) used for confirming the measurement model and research hypothesis assumes testing the extent to which a hypothesized model provides an appropriate characterization of the collective relationships among research variables. For this purpose, researchers must assess the “fit” between the model and the sample’s data.
There is no data on model fit in the results section of the article. CFI, TLI, GFI, or RMSEA indices are recommended for model equivalence-testing to test and compare goodness of fit for structural equation models (Peugh & Feldon, 2020). Further analysis of variable interconnections in Table 5: Hypothesis results (p.13) is not informative without analysing model fit.

Using SMART-PLS for SEM can be justified by the small size of the sample. Flexibility in model building allows a more flexible approach to model building and enables researchers to modify and refine models iteratively. However, SMART-PLS is more focused on exploratory research and with limited support for advanced statistical techniques such as mediation and moderation analysis. Due to its reliance on bootstrapping, SMART-PLS generally has lower statistical power compared to AMOS.
Discussing the choice of SMART-PLS, it is worth interpreting this study as exploratory research that requires further investigation of model quality.
Moreover, discussing this in the results section of the article does not seem to be relevant. This probably should be done in the limitations section of the article.

**Response:** We acknowledge this review’s extensive and helpful remarks on the application of structural equation modeling (SEM) and the absence of model fit indices in the results section. Although SMART-PLS does not offer traditional model fit indices like CFI, TLI, GFI, or RMSEA as is normally provided in covariance-based SEM techniques like AMOS, we recognize this. We now explicitly address this limitation in the section Limitations and Future Research Directions in the revised manuscript. Given the exploratory nature of this study and moderate sample size (N = 392), the choice of SMART-PLS was made. Although SMART-PLS helped us to refine our model iteratively, and permitted the analysis of mediation and moderation effects, it did not permit the inclusion of traditional model equivalence-testing metrics. To overcome this limitation, we have clarified in the revised manuscript that future studies should gather larger datasets and use advanced SEM techniques that allow one to test model equivalence and include fit indices. The use of this approach will facilitate a robust evaluation of the proposed relationships and migration from exploratory to confirmatory research.

In addition, we have reworked the manuscript by moving some methodological discussion related to SMART-PLS from the results section to the Limitations section. This revision brings the results section back to only findings like in any standard reporting practice. We thank the reviewer for suggesting we move these discussions to other locations because it brings greater clarity and structure to the manuscript. We believe these changes resolve the reviewer’s concerns without compromising the rigor or integrity of the study, and look forward to building upon this foundation in future work. Once again, your valuable feedback has greatly added quality to our manuscript.

4. Proofreading.
Addressed.

**Reviewer #3: Dear Author's ,**

I wanted to let you know that the revisions to your article, "Sustainable Care: How CSR Shapes Wellbeing in Healthcare Organizations in Beijing, Shanghai, and Guangzhou," have significantly improved the overall quality of the research. You've effectively addressed all my suggestions, and the article now presents a clearer and more structured link between CSR initiatives, employee burnout, and well-being. The incorporation of sustainable development goals (SDGs) in your discussion has further enriched the paper's relevance to current global health priorities.

The inclusion of mediators such as happiness and psychological safety, along with altruism as a moderator, adds depth to the analysis and supports your findings on the role of CSR in reducing burnout. The article now makes a strong contribution to the field, emphasizing the importance of CSR strategies in healthcare for achieving sustainable development and enhancing employee well-being.

Wis you good luck.

**Response:** Dear Reviewer, we sincerely thank you for the positive feedback and for liking our revised efforts. Certainly, your valued feedback helped us in improving the overall quality of our manuscript prior to publishing.
